# Intrinsic and extrinsic factors related to pathogen infection in wild small mammals in intensive milk cattle and swine production systems

**DOI:** 10.1371/journal.pntd.0005722

**Published:** 2017-06-30

**Authors:** Rosario Lovera, María Soledad Fernández, Jens Jacob, Nidia Lucero, Gabriel Morici, Bibiana Brihuega, María Isabel Farace, Jorge Caracostantogolo, Regino Cavia

**Affiliations:** 1 Departamento de Ecología, Genética y Evolución, Facultad de Ciencias Exactas y Naturales, Universidad de Buenos Aires and Instituto de Ecología, Genética y Evolución de Buenos Aires (IEGEBA), UBA-CONICET, Cdad. Autónoma de Buenos Aires, Argentina; 2 Federal Research Centre for Cultivated Plants – Julius Kuehn Institute, Vertebrate Research, Münster, Germany; 3 Administración Nacional de Laboratorios e Institutos de Salud “Dr. Carlos G. Malbrán” (ANLIS), Cdad. Autónoma de Buenos Aires, Argentina; 4 Área de Parasitología, Instituto de Patobiología, Instituto Nacional de Tecnología Agropecuaria (INTA Castelar), CICVyA, Buenos Aires, Argentina; 5 Laboratorio del Leptospirosis, Referencia OIE, Instituto de Patobiología, Instituto Nacional de Tecnología Agropecuaria (INTA Castelar), CICVyA, Buenos Aires, Argentina; School of Veterinary Medicine University of California Davis, UNITED STATES

## Abstract

**Background:**

Understanding the ecological processes that are involved in the transmission of zoonotic pathogens by small mammals may aid adequate and effective management measures. Few attempts have been made to analyze the ecological aspects that influence pathogen infection in small mammals in livestock production systems. We describe the infection of small mammals with *Leptospira* spp., *Brucella* spp., *Trichinella* spp. and *Cysticercus fasciolaris* and assess the related intrinsic and extrinsic factors in livestock production systems in central Argentina at the small mammal community, population and individual levels.

**Methodology/Principal findings:**

Ten pig farms and eight dairy farms were studied by removal trapping of small mammals from 2008 to 2011. Each farm was sampled seasonally over the course of one year with cage and Sherman live traps. The 505 small mammals captured (14,359 trap-nights) included three introduced murine rodents, four native rodents and two opossums. *Leptospira* spp., anti-*Brucella* spp. antibodies and *Trichinella* spp. were found in the three murine rodents and both opossums. *Rattus norvegicus* was also infected with *C*. *fasciolaris*; *Akodon azarae* and *Oligoryzomys flavescens* with *Leptospira* spp.; anti-*Brucella* spp. antibodies were found in *A*. *azarae*. Two or more pathogens occurred simultaneously on 89% of the farms, and each pathogen was found on at least 50% of the farms. Pathogen infections increased with host abundance. Infection by *Leptospira* spp. also increased with precipitation and during warm seasons. The occurrence of anti-*Brucella* spp. antibodies was higher on dairy farms and during the winter and summer. The host abundances limit values, from which farms are expected to be free of the studied pathogens, are reported.

**Conclusions/Significance:**

Murine rodents maintain pathogens within farms, whereas other native species are likely dispersing pathogens among farms. Hence, we recommend preventing and controlling murines in farm dwellings and isolating farms from their surroundings to avoid contact with other wild mammals.

## Author summary

Some rodents and opossums can transmit zoonotic diseases, thereby causing economic losses and sanitary problems, including damage to livestock production systems worldwide. Identifying the factors affecting pathogen transmission will contribute to a better understanding of the mechanisms involved. In this study, the infection of four zoonotic pathogens in small mammals captured on intensive pig and dairy farms in central Argentina and potential environmental drivers of those infections were studied. Seven rodent and two opossum species were trapped on these farms. Murine rodents and opossums were infected with *Leptospira* spp. and *Trichinella* spp. and carried anti-*Brucella* spp. antibodies. *Rattus norvegicus* was also infected with *C*. *fasciolaris*, and some native rodents were infected with some of the studied pathogens. Each pathogen occurred on at least 50% of the farms, and two or more pathogens occurred on 89% of the farms. These results reflect a high circulation of zoonotic pathogens relevant to public health on intensive livestock farms. Moreover, all pathogen infections increased with host abundances. Murine rodents maintain pathogens within farms, whereas other native species are likely dispersing pathogens among farms.

## Introduction

Extrinsic factors such as climatic conditions and environmental characteristics [1–3], and intrinsic factors such as host characteristics [4, 5] influence pathogen transmission. Identifying the factors that affect pathogen transmission at different levels (i.e., small mammal community, population or individual level) will contribute to a better understanding of the mechanisms of zoonosis transmission, because factors may have a different effect at different levels. Understanding the ecological processes involved in the transmission of zoonotic pathogens is important for designing adequate management actions [6]. The constant availability of food, water and shelter for wildlife, as well as livestock over-crowding and poor hygiene, make livestock production systems particularly attractive to wildlife [7]. Some rodent and opossum species carry several zoonotic pathogens [8, 9], such as bacterial, viral, protozoonotic and helminthic pathogens [i.e., 10–15]. These mammals also cause economic losses in agriculture and other production systems [16–18]. The magnitude of damage and the health risk caused by rodents and opossums have traditionally led to mechanical and chemical control measures. However, control actions are sometimes ineffective in the long term because they are poorly timed or inadequate and ecological information about the species to be managed is lacking [16, 19]. Leptospirosis, brucellosis and trichinosis are among the most important zoonotic infections worldwide [20–22]. They are endemic in Argentina [23–25] and represent a risk for personnel and lead to production losses in cattle [26–28].

There are at least nine pathogenic *Leptospira* species. Humans and other animals can be infected if they are exposed to urine [29]. Murine rodents (*Rattus norvegicus*, *R*. *rattus* and *Mus musculus*) are mainly the permanent hosts and important carriers of pathogenic leptospires [26, 30], but there are many other host species [9, 31]. *Leptospira interrogans* and *L*. *borgpetersenii* are present in rodents and opossums throughout the world including Argentina [i.e., 12, 31–35]. The prevalence of *L*. *interrogans* in rats increases with age [36, 37] and is more prominent in females [36]. Seroprevalence in pigs is higher at high precipitation, high temperature and high relative humidity, which indicates the influence of climate variables in the presence of this pathogen [38].

Several species of *Brucella* spp. occur in wildlife, domesticated livestock and humans [39–41]. Each *Brucella* species has a preferential host and specific virulence. Brucellosis is acquired by direct contact, via aerosols from infected blood, placenta, fetuses or uterine secretions or by consuming infected animal products such as milk. Brucellosis usually leads to abortion, which may result in economic losses in animal production [42]. In Argentina, this disease is present in pigs [43, 44] and cows [45, 46], although it is more common in cows [47]. *Brucella* spp. has been isolated from several rodent species and opossums worldwide [48–50], but the factors that influence infection are unclear. In Argentina, there have not been major surveys to identify wild mammals as *Brucella* spp. hosts [47, 51].

The nematode *Trichinella spiralis* is the etiological agent of trichinosis that is transmitted and maintained in a domestic cycle that includes pigs, rats, mice, and other synanthropic and wild mammals [52, 53]. Humans are accidental hosts that acquire trichinosis through the consumption of undercooked pork meat infected with *T*. *spiralis* encysted larvae. In Argentina, trichinosis is an important zoonosis for public health because outbreaks occur [53]. Although the role of rodents in the transmission cycle is under debate [54, 55], evidence of a high *T*. *spiralis* prevalence in Argentinian production systems with evidence of wildlife suggests the circulation of this pathogen between wild and domestic animals [23, 56]. Most prior studies have focused on infection patterns in rodents [i.e., 56, 57–59], but works on related factors are scarce [i.e., 60, 61].

*Cysticercus fasciolaris* is the infective larval stage of the metacestode of the cat tapeworm *Taenia taeniaeformis*. Adults are found in the small intestine of their definitive hosts, such as felids, dogs, foxes and sporadically humans [62, 63]. Rodents, insectivores, lagomorphs and occasionally humans are the intermediate hosts of *C*. *fasciolaris* [62]. Occasionally, human cases have been reported from Argentina and other countries [Miyazaki et al., 1991 and Ekanayake et al., 1999 in 64]. Previous studies have shown that the prevalence of helminthes (including *T*. *taeniaeformis*) in rodents is influenced by the age, sex, season and habitat [65, 66].

The Pampas region accounts for approximately 99% of milk [67] and 70% of livestock swine production in Argentina [68]. Screening studies in this region showed the presence of *L*. *interrogans*, *T*. *spiralis*, *Salmonella enterica* and hantaviruses in rodents [58, 69–71] and *T*. *spiralis*, *L*. *interrogans* and *S*. *enterica* in opossums [12, 69, 72]. However, little is known about the factors that influence pathogen infection, the importance of wildlife and its role in the epidemiology of the transmission of zoonoses on farms where contact between wild and domestic animals is likely. Environmental drivers of pathogen prevalence and, hence, the transmission risk in small mammal hosts in such systems are unknown. Knowledge about these drivers may aid effective sanitary and control measures.

The aim of this study was to describe the infection with *Brucella* spp., *Leptospira* spp., *Trichinella* spp. and *C*. *fasciolaris* in small mammals and to assess related intrinsic and extrinsic factors on intensive pig and dairy farms in central Argentina at three levels for the small mammals: community, population and individual. We hypothesized that infection depends on both extrinsic and intrinsic factors:

1. Not all small mammal species are equally infected with *Brucella* spp. or *Trichinella* spp. We expected diet to affect *Trichinella* spp. infection in small mammals because the consumption of meat is required for infection. We predicted that species habitat preferences affect *Brucella* spp. and *Trichinella* spp. infection because species more associated with livestock (i.e., murine rodents) are most likely to be infected.
2. The infection of pathogens is density-dependent. We expected that infection with pathogens would be higher with higher small mammal abundances at the community and population levels.
3. The infection of pathogens differs between production systems. We predicted that *Trichinella* spp. infection would be higher on pig farms, but that *Brucella* spp. prevalence would be higher on dairy farms.
4. Weather is linked to infection risk. We expected that precipitation and temperature would positively affect infection with *Leptospira* spp.
5. The infection of pathogens depends on the age of the individuals. We assumed that the occurrence of *Trichinella* spp. and *Leptospira* spp. would be higher in older rather than young individuals.

## Materials and methods

### Ethics statement

Trapping, handling and euthanasia were done following the procedures and protocols approved by the Argentine Law for Animal Care 14346, the Argentinean Society for Mammalian Studies [75], the American Society of Mammalogists [76] and the Ethics Committee for Research on Laboratory Animals, Farm and Obtained from Natures of National Council of Science and Technical Research (CONICET; resolution 1047, section 2, annex II), and subsequently the National Agency for the Promotion of Science and Technology of Argentina (ANPCYT PICT-2007-01432) and the National Council of Scientific and Technical Research (CONICET PIP1410-2009-11). No endangered species were involved in this study.

### Study area and farms description

The study took place in the counties of General Las Heras, Marcos Paz, San Andrés de Giles and Exaltación de la Cruz (northeast of Buenos Aires province, Argentina (34° S, 58.5° W)), on pig and dairy farms. The geographic area belongs to the Rolling Pampa [73] where the climate is temperate with a mean annual temperature of 17.4°C and a mean annual precipitation of 1,014 mm [74]. Farms were surrounded by crops, grasslands and pastures for livestock. The abundant availability of food and water favors the presence of small mammals that access livestock feed [for a detailed description of the farms, see 7].

### Trapping procedure

Trapping surveys were performed on 10 pig farms and eight dairy farms from spring 2008 to spring 2011. Each farm was sampled during one year for four consecutive seasons. Five habitats were surveyed on each farm (when possible): 1. animal sheds (dairy and pig sheds): structures in which pigs or cows were present; 2. food storage sheds or silos; 3. human buildings: dwellings with high human activity not used to store food such as houses, machinery sheds, warehouses and offices; 4. vegetated areas around dwellings; and 5. drainage channels with adjacent dirt mounds with tall herbaceous vegetation. Not all habitats were present on all farms.

Small mammals were captured using cage live-traps (15x16x31 cm) and Sherman traps (8x9x23 cm). Both types of traps were set adjacent to each other every 10m along trap-lines of 50–100 m with 1–3 replicates per habitat. In each trapping session, the location of the traps was the same. Traps were active for three consecutive nights and checked daily in the morning. Captured individuals were identified to species and sex. Individuals were humanely sacrificed to collect tissue samples after a deep anesthesia with an intramuscular injection of ketamine-acepromazine (rodents by cervical dislocation and opossums with an overdose of isofluorane).

### Sample collection and pathogen detection

For *Leptospira* spp. detection, two types of analyses were conducted. First, urine samples (until spring 2009) and kidney tissue smears (until spring 2010) from each individual were placed in EMJH (Ellinghausen-Mccullough-Johnson-Harris) liquid medium. These samples were incubated at 30°C adding 5-fluorouracil aseptically as cytostatic and examined weekly by dark-field microscopy for 60 days. Second (after spring 2010), aliquots of renal tissue were incubated in EMJH and Fletcher semi-solid medium at 30°C and examined regularly every 15 days by dark-field microscopy during six months. Direct immunofluorescence (DI) was performed to evaluate the presence of leptospires in imprints from the kidney [77]. Molecular characterization of the isolated strains was carried out by Multiple-Locus Variable-Number Tandem Repeat (VNTR) Analysis (MLVA), a Polymerase Chain Reaction (PCR)-based method to identify the serovars of *L*. *interrogans*, using two sets of primers flanking a total of 12 loci [78]. For *L*. *kirschneri* and *L*. *borgpetersenii*, the primer pairs proposed by Salaün *et al*. [79] were used to flank the VNTRs: 4bis, 7bis, 10bis, Lb4 and Lb5. To compare the repetition codes obtained in the molecular characterization the Pavan *et al*. [35] criteria were used.

For *Brucella* spp. detection, blood samples were collected by cardiac puncture and serum was obtained. For the screening of antibodies against smooth *Brucella* spp. (*B*. *melitensis*, *B*. *abortus* and *B*. *suis*) the buffered plate agglutination test (BPAT) and the Rose Bengal test (RBT) were run. The serum agglutination test (SAT) and the 2-mercapto-ethanol test (MET) were used to confirm the results [80]. For the detection of antibodies against rough *Brucella* spp. (*B*. *ovis* and *B*. *canis*), the rapid slide agglutination test (RSAT) was used for screening [81]. For bacteriological assays, the other kidney, spleen and liver were removed from each individual and portions of these tissues were cut with scissors and smeared on two plates of BDS (*Brucella* Broth BBL + Bacto Agar + filter sterilized equine serum 3%) and on two plates of BDA (*Brucella* Broth BBL + Bacto Agar + antibiotics) [82]. Plates were incubated at 36±1°C with 5–10% CO_2_ for 14 days.

For *Trichinella* spp. detection, the tongue, diaphragm, intercostal, cheek and/or leg muscles of each captured individual were removed, and all muscle samples were artificially digested in a solution of 1% HCl and 1% pepsin [83]. All larvae isolated were morphologically identified by the presence of stichosome, which is the main morphological characteristic diagnostic of the genus. *Trichinella* larvae isolated from opossums were identified to the species level by nested multiplex PCR and confirmed by sequencing [for details, see 72].

We recorded individuals with parasitic larval capsules of metacestodes that were visible on the liver surface. Some of these capsules were preserved for morphological and molecular identification [84]. Eye lenses of each individual were removed and preserved in 10% formalin to use its dry weight as an age indicator [85].

### Data analysis

The prevalence of the studied pathogens was compared among small mammal species using an independence test [86]. When significant differences were observed, we subdivided the contingency tables according to Zar [86]. When more than one technique was used to analyze the presence of a pathogen in an individual, it was considered positive if at least one of those techniques yielded a positive result.

Trap success was determined for total small mammals and of each species on each farm and in each season [87]. On the individual level, trap success was also calculated for each habitat, farm and season. Because of their size, some small mammal species are captured only in the cage (rats, caviids and opossums) or Sherman traps (mice). For these species, trap success was estimated by considering only the relevant trap type. Temperature data were obtained from the meteorological station of Ezeiza [88] and precipitation data from the meteorological stations of Solis, Lobos, Navarro and San Andrés de Giles [89], Buenos Aires province.

On the community level, we analyzed the effects of environmental factors on the occurrence of each pathogen. Pathogen presence was assumed if at least one infected individual was captured on a farm during a trapping session. The type of production system (pig or dairy farm), season, monthly mean temperature (°C), accumulated precipitation in the previous month (dm) and in the last six months (dm) prior to the captured date, small mammal abundance, species richness and interactions of these variables were considered in the analysis.

On the population level, we studied the occurrence and prevalence of pathogens in each population and we analyzed the effects of environmental factors. All environmental parameters considered at the community level, host population abundance and interactions of these variables were included. We considered a pathogen to be present in a population (on a farm in each season) if at least one individual was infected.

On the individual level, we analyzed the effect of all environmental parameters considered at the community and population levels for each pathogen. We also tested for the effects of habitat, sex and age (in months) and their interactions.

For these analyses, Generalized Linear Mixed Models (GLMMs) with binomial error structure, a logit-link function and the Laplace approximation method were used [90]. Farm was always included in the model as a random effect because farms were sampled repeatedly (in each season). When the random effect did not improve the model (based on the change of deviance between models with and without the random factor), the factor farm was removed and Generalized Linear Models (GLMs) were used. On the population and individual levels, the analyses were conducted for each host species separately. For all GLMs and GLMMs analyses the forward stepwise regression procedure was used and the simplest significant models were reported [90]. Variables that were highly correlated (*r*_Pearson_>|0.6| or *p*<0.01) to variables that had already been included in the model were discarded. Collinearity among all predicted variables was assessed with the Variance Inflation Factors (VIFs) [91]. If any VIF value was much larger than 5, which indicated multicollinearity, the variable or interaction was removed, the VIFs were recalculated and the process was repeated until all the VIFs were smaller than the preselected threshold [91]. When more than one candidate model was found, we employed the Akaike Information Criterion (AIC) for model selection and we only report models with ΔAIC<7 [92]. For the occurrence models, the accuracy measures Kappa index (K), sensitivity, specificity and proportion of correct classifications (PCC) were calculated [93, 94]. GLMMs, VIFs and the accuracy measures were conducted using the *lme4* [95], *car* [96] and *PresenceAbsence* package [97] in the R software [98], respectively.

## Results

### Pathogen occurrence and prevalence

We caught 444 rodents of seven species (*R*. *norvegicus*, *R*. *rattus*, *M*. *musculus*, *Akodon azarae*, *Calomys laucha*, *Oligoryzomys flavescens* and *Cavia aperea*) and 61 opossums of two species (*Didelphis albiventris* and *Lutreolina crassicaudata*) with a sampling effort of 7,333 Sherman trap-nights and 7,026 cage live trap-nights (Table 1). All species were captured in both production systems.

**Table 1:** Prevalence (%) and number of individuals (n) in serological, bacteriological and parasitological analyses of pathogens in seven rodent and two opossum species captured on 18 livestock farms in central Argentina from 2008 to 2011. Numbers in the last row refer to the percentage of farms where the pathogen occurred.

		*Leptospira* spp.		Anti-*Brucella*		*Trichinella* spp.		Metacestodes*	
	Total	%	n	%	N	%	n	%	n
**Rodentia**									
Murines									
*R*. *norvegicus*	281	24.0 ^A^	179	24.9† ^A^	241	3.0 ^B^	266	39.2	278
*R*. *rattus*	17	18.2 ^A^	11	7.1 ^B^	14	17.7 ^A^	17	0	17
*M*. *musculus*	86	13.9 ^A^	72	3.2† ^B^	62	2.6 ^B^	78	0	83
Sigmodontines									
*A*. *azarae*	41	18.2 ^A^	33	3.0 ^B^	33	0.0 -	36	7.3	41
*C*. *laucha*	6	0.0 -	3	0.0 -	6	0.0 -	5	0	6
*O*. *flavescens*	7	50.0 -	2	0.0 -	6	0.0 -	7	0	7
Caviid									
*C*. *aperea*	6	0.0 -	4	0.0 -	6	0.0 -	6	0	6
**Didelphimorphia**									
Opossums									
*D*. *albiventris*	41	8.0 ^A^	25	5.3† ^B^	38	7.5 ^B^	40	0	41
*L*. *crassicaudata*	20	13.3 ^A^	15	12.5 ^B^	16	5.3 ^B^	19	0	20
** Individuals**	**505**		**344**		**422**		**474**		**499**
** Farms**	**18**	77.8	18	66.7	18	50.0	18	72.2	18

Upper case letters refer to significant differences among small mammal species (*p* < 0.05).

^-^ Species excluded due to small sample size.

^†^ One *R*. *norvegicus*, one *D*. *albiventris* and one *M*. *musculus* were positive for smooth *Brucella* spp. antibodies, from which two were positive according to SAT and 2ME.

* Metacestodes in *R*. *norvegicus* were *C*. *fasciolaris*, but in *A*. *azarae*, they could not be determined (see text).

The three murines and both opossums were infected with pathogenic *Leptospira* spp., carried anti-*Brucella* spp. antibodies and *Trichinella* spp., whereas none of these pathogens were present in *C*. *laucha* and *C*. *aperea* (Table 1). *Akodon azarae* and *O*. *flavescens* were infected with *Leptospira* spp. and the former also carried anti-*Brucella* spp. antibodies (Table 1). Metacestodes of tapeworms were found encysted in the liver of *R*. *norvegicus* and *A*. *azarae* (Table 1). All capsules were morphologically consistent with *C*. *fasciolaris* [99]. Based on both morphological and molecular data, the metacestode in *R*. *norvegicus* was *C*. *fasciolaris* (Table 1) [for more information, see 84]. In *A*. *azarae* the metacestode species was not identified, but it showed the same characteristics as in *R*. *norvegicus*, and *C*. *fasciolaris* was previously reported to have infected *A*. *azarae* [100]. Because we identified metacestodes to the species level only in *R*. *norvegicus*, we refer to metacestodes at the community level and to *C*. *fasciolaris* at the population and individual levels.

Pathogenic *Leptospira* spp. was detected in the three murines, the sigmodontines *A*. *azarae* and *O*. *flavescens*, and in both opossums (Table 1) at a similar prevalence (*χ*^*2*^ = 6.32, *d*.*f*. = 5; *p* = 0.277; Table 1). Of the bacteria isolated from 15 of the 66 positive individuals, *L*. *interrogans* serovar Icterohaemorrhagiae was present in 10 *R*. *norvegicus* and one *M*. *musculus*; *L*. *borgpetersenii* in one *L*. *crassicaudata*, one *O*. *flavescens* and one *R*. *rattus*; and *L*. *kirschneri* in one *R*. *norvegicus*.

Anti-*Brucella* spp. antibodies were detected in all murines, *A*. *azarae* and both opossums (Table 1). Of 422 samples analyzed, 65 carried rough *Brucella* spp. antibodies (30 weakly positive) and three carried smooth *Brucella* spp. antibodies (Table 1). More *R*. *norvegicus* carried anti-*Brucella* spp. antibodies than the other two murines, opossums and *A*. *azarae* (*χ*^*2*^ = 27.75, *d*.*f*. = 1; *p*<0.001; Table 1). Despite the detection of antibodies, the bacterium *Brucella* spp. was not isolated from any of the 474 individuals analyzed (271 *R*. *norvegicus*, 17 *R*. *rattus*, 71 *M*. *musculus*, 41 *A*. *azarae*, 6 *C*. *laucha*, 7 *O*. *flavescens*, 6 *C*. *aperea*, 36 *D*. *albiventris* and 19 *L*. *crassicaudata*).

*Trichinella* spp. larvae were detected in the three murines and both opossums (Table 1). *Rattus rattus* was more frequently infected with *Trichinella* spp. than were the other four species (*χ*^*2*^ = 8.44, *d*.*f*. = 1; *p* = 0.004, Table 1). *Trichinella spiralis* larvae were isolated from both opossums for the first time in these species [72].

All pathogens were present in both production systems and each pathogen was present on at least 50% of the farms (Table 1). *Leptospira* spp. showed the highest proportion of farms infected and *Trichinella* spp. the lowest (Table 2). We found multiple infections with four pathogens on six farms, and on 16/18 farms, we found more than one pathogen (Table 2). We found only anti-*Brucella* spp. antibodies on one pig farm, but no pathogens were present on one dairy farm (Table 2).

**Table 2:** Occurrence of each pathogen (+) and pathogen richness on each of the 18 livestock farms in central Argentina from 2008 to 2011. Occurrence was assumed if at least one individual was positive at that farm. Prop: Proportion of farms in which each pathogen occurred over the 18 studied livestock farms. P: pig farm and D: dairy farm.

Type of farm	P	P	P	D	D	D	P	P	D	P	P	P	D	D	P	D	P	D	
Pathogen richness	4	4	4	4	4	4	3	3	3	2	2	2	2	2	2	2	1	0	
																			Prop
*Leptospira* spp.	+	+	+	+	+	+	+	+	+	+	+	+	+	+					**14/18**
Metacestodes	+	+	+	+	+	+	+	+	+	+	+				+	+			**13/18**
anti-*Brucella* spp.	+	+	+	+	+	+	+	+	+						+	+	+		**12/18**
*Trichinella* spp.	+	+	+	+	+	+						+	+	+					**9/18**

### Extrinsic and intrinsic factors

Due to sample size, population and individual level models were conducted only for *R*. *norvegicus*. Analyses in *M*. *musculus* were restricted to *Leptospira* spp. Age was estimated for these two murines [101, 102].

Precipitation during the previous month was correlated with both the monthly mean temperature and the precipitation in the last six months (*r*_Pearson_ = 0.40 and *r*_Pearson_ = 0.72, respectively; *p*<0.001 for both cases). Small mammal abundance was highly correlated with *R*. *norvegicus* population abundance and small mammal species richness (*r*_Pearson_ = 0.83 and *r*_Pearson_ = 0.48, respectively; *p*<0.001 for both cases).

The occurrence of *Leptospira* spp. at the community level increased when small mammal abundance increased (Table 3, S1 Appendix). Occurrence and prevalence in *R*. *norvegicus* populations depended on the synergistic effect of monthly precipitation and small mammal abundance. The infection in *R*. *norvegicus* increased in warm seasons or human buildings when precipitation increased (Table 3, S1 Appendix). None of the parameters considered explained pathogen occurrence and prevalence in *M*. *musculus* at the population or individual level.

**Table 3:** Summary of the simplest Generalized Linear Models for the pathogens found on the 18 livestock farms in central Argentina from 2008 to 2011 at the three studied levels. At the small mammal community level, explaining the occurrence of each pathogen taking into account all the individuals captured on each farm and trapping session; at the population level, explaining the occurrence and prevalence of each pathogen in *R*. *norvegicus* populations taking into account only *R*. *norvegicus* individuals on each farm and trapping session; and at the individual level, explaining infection of each pathogen in *R*. *norvegicus* individuals. SM AB: small mammal abundance; PP and PP6: accumulated monthly precipitation and accumulated precipitation in the last six months, respectively; Type: type of productive system (dairy or pig farm); Temp: monthly mean temperature (°C); *Rn*: *R*. *norvegicus*; *d*.*f*.: residual degrees of freedom; % dev: percentage of deviance explained by the model; K: Kappa index; Sens: sensitivity, Spec: specificity; PCC: proportion of correct classifications.

							Accuracy measures				
	Response variable	Model or models	*d*.*f*.	AIC	Null AIC	% dev	K	PCC	Sens	Spec	Cut point
***Leptospira* spp.**											
Community level:	Occurrence	SM AB	54	73.94	79.56		0.39	0.70	0.72	0.67	0.44
Population level:	*Rn* occurrence	PP*SM AB	34	53.14	54.68		0.32	0.66	0.58	0.74	0.47
	*Rn* prevalence	PP*SM AB	34	87.99	98.91	26.98					
Individual level:	*Rn* infection	Season*PP6	161	193.19	193.70		0.21	0.69	0.44	0.78	0.34
		Habitat*PP6	161	189.41	193.70		0.28	0.75	0.40	0.87	0.40
**Anti–*Brucella* spp.**											
Community level:	Occurrence	Season + SM AB	52	60.29	78.88		0.56	0.79	0.70	0.86	0.44
		Type*SM AB	53	62.65	78.88		0.57	0.81	0.52	1.00	0.61
Population level:	*Rn* occurrence	SM AB	40	42.39	59.36		0.65	0.83	0.72	0.92	0.48
	*Rn* prevalence	Season	38	110.12	117.32	17.77					
Individual level:	*Rn* infection	Season + Habitat*Type + Habitat*SM AB	200	241.71	250.69		0.36	0.69	0.79	0.66	0.24
***Trichinella* spp.**											
Community level:	Occurrence	SM AB	59	63.90	72.20		0.46	0.82	0.44	0.96	0.48
Population level:	*Rn* occurrence	SM AB	44	35.24	41.23		0.56	0.91	0.43	1.00	0.54
	*Rn* prevalence	Null model									
Individual level:	*Rn* infection	Age*Sex	262	73.42	73.80		0.08	0.74	0.63	0.75	0.04
**Metacestodes**											
Community level:	Occurrence	SM AB	61	67.42	88.94		0.58	0.79	0.73	0.85	0.39
Population level:	*Rn* occurrence	SM AB	45	47.70	64.56		0.53	0.77	0.72	0.83	0.54
	*Rn* prevalence	Type + PP6	44	144.97	150.00	9.90					
		SM AB	45	144.24	150.00	8.50					
Individual level:	*Rn* infection	Age*SM AB + Habitat*Type + Temp.	264	289.05	374.34		0.53	0.78	0.62	0.89	0.51

The occurrence of *Brucella* spp. antibodies at the community level and at the *R*. *norvegicus* population and individual levels increased with the increase of small mammal abundance, being higher in the winter and summer for the community and individual levels, and higher on dairy farms at the community level (Table 3, S1 Appendix). Seroprevalence at the *R*. *norvegicus* population level was also higher in the winter and summer. *Brucella* spp. antibodies in *R*. *norvegicus* individuals also increased with small mammal abundance, but this occurred differently among habitats and type of production system (Table 3, S1 Appendix).

The occurrence of *Trichinella* spp. at the community and *R*. *norvegicus* population level increased with the increase of small mammal abundance, but none of the parameters considered explained pathogen prevalence in *R*. *norvegicus* populations (Table 3). The individual infection in *R*. *norvegicus* increased with age in females (Table 3, S1 Appendix).

The occurrence and prevalence of metacestodes at the community level and of *C*. *fasciolaris* in *R*. *norvegicus* populations increased with the increase of small mammal abundance (Table 3). Additionally, the prevalence was higher on dairy farms and decreased with precipitation in the last six months (Table 3). The infection of *R*. *norvegicus* individuals increased with the increase of temperature, with increasing age as the small mammal abundance increased, and was more frequent on dairy farms but differently among habitats (Table 3, S1 Appendix).

Most of the occurrence models had a moderate to substantial agreement, but some of them had a fair agreement (Table 3) [94]. Only *Trichinella* spp. occurrence at the individual level model had a slight agreement; however PCC, sensitivity and specificity indicated a better agreement (Table 3) [94].

Because of abundance effects on occurrence, we estimated abundance limit values (individuals/100*trap-nights) for each pathogen both at the community and *R*. *norvegicus* population level; above these levels, pathogens are expected to occur (Table 4). These values are also relevant for management actions because they would represent the acceptable small mammal infestation level in these production systems.

**Table 4:** Limit values of: small mammal abundance at the small mammal community level and *Rattus norvegicus* abundance at the population level for each pathogen, estimated with 18 livestock farms of central Argentina from 2008 to 2011. Abundances were estimated by its trap success: individuals/100*trap-nights. Since host abundance on farms explained pathogen occurrence, above the reported abundance values, each pathogen is expected to occur on a farm.

Pathogen	Small mammal abundance	*R*. *norvegicus* abundance
*Leptospira* spp.	7.6	7.0†
anti-*Brucella* spp.	4.5*	11.0
*Trichinella* spp.	18.6	26.3
Metacestodes	7.9	5.5

^†^ Estimated with monthly average precipitation.

* Estimated for winter.

## Discussion

We found four pathogens that were highly relevant to public health and occurred regularly on more than 50% of the farms in both production systems and in several mammalian species. Farming practices favor pathogen survival and pathogen circulation among small mammal species and among individuals. The high community density of small mammal abundance promotes transmission of pathogens to susceptible small mammals and potentially livestock and humans. Studies involving more than one mammalian host species and more than one pathogen in production systems are rare [69, 103, 104], and most studies were restricted to pathogen prevalence [i.e., 32, 48, 60, 69, 105, 106]. Generating such information is vital for managing overabundant small mammal populations to enhance food security and general hygiene in production systems and for minimizing pathogen transmission from wildlife reservoirs to humans and livestock.

*Leptospira* spp. was present with a similar prevalence in murines and opossums, as previously reported for rodents [106, 107], which indicated that several small mammal species have the potential to maintain this pathogen in the farm environment. As expected, *Trichinella* spp. and anti-*Brucella* spp. antibodies were more prevalent in *Rattus* species than in host species inhabiting habitats remote from livestock. *Rattus norvegicus*, which was the dominant species in both systems [7], carried all pathogens considered. The prevalence of *L*. *interrogans* in this species is highly variable even at local scales [105, 108–111]. According to our results, the prevalence of this bacterium was affected by small mammal abundance, weather and habitat. There is limited information related to the prevalence/seroprevalence of brucellosis in wildlife reservoirs [112], although some studies have reported the isolation and transmission of *Brucella* spp. in rodents [113] on farms with infected livestock [114–116]. The prevalence of *Trichinella* spp. in *R*. *norvegicus* worldwide varies between 0–42.4% [56, 58, 60, 61, 69], whereas the prevalence for *C*. *fasciolaris* varies between 30–40% [60, 117, 118], as also reflected in this study.

As hypothesized, pathogen infections increased with small mammal abundance confirming earlier findings [119, 120]. Occurrence patterns were similar at the community and *R*. *norvegicus* population level which may have been due to the dominance of this species in both production systems. Consequently, it remains unclear whether small mammal abundance or *R*. *norvegicus* abundance determines pathogen infection. Moreover, regulating the abundance of small mammals that are most likely to carry pathogens can help to prevent transmission [3, 121].

*Rattus rattus*, *M*. *musculus* and both opossums carried three of the four studied pathogens. The finding of opossums with *T*. *spiralis* for the first time [72] supported a diet study on these livestock farms, where we found that rodents are a part of the opossums diet [122], consistent with our hypothesis. *Akodon azarae* carried all the pathogens except *Trichinella* spp., as expected, because it is a folivorous-insectivorous species. Neither *C*. *laucha*, *O*. *flavescens* nor *C*. *aperea* carried *Trichinella* spp., which may reflect their diets that are based on plants and insects. In the last three species we did not find pathogens, with the exception of one *O*. *flavescens* that was positive for *Leptospira* spp. Metacestodes were reported in *R*. *rattus*, *M*. *musculus* and *O*. *flavescens*, suggesting a relationship between *C*. *fasciolaris* infection to the presence of *R*. *norvegicus* in rodent communities [123, 124].

An increase in species diversity could increase the transmission risk and prevalence of ubiquitous pathogens, known as the “amplification effect” [125]. Abundance was a key factor in pathogen infection. However, due to the positive correlation between species richness and small mammal abundance, it is not possible to separate these two effects. The “dilution effect” postulates that an increase in species diversity reduces the transmission of host specific pathogens [125]. Our results support the “amplification effect” may be because the pathogens considered are not host-specific and we found several mammalian species infected with these pathogens, such as *Leptospira* spp. [126].

As we expected for *Leptospira* spp., accumulated precipitation influenced small mammal infection, consistent with previous work [22, 127]. Precipitation mattered despite the continuous presence of water bodies in these systems which are accessed by livestock and small mammals. We also found that seasonality affects the individual infection in *R*. *norvegicus*. Leptospires survival outside the host is favored by humid and warm conditions [128], as reflected in both production systems despite some differences between them [for a detailed description, see 7]. However, we found differences in the individual infections in *R*. *norvegicus* among habitats, consistent with Cosson *et al*. [107].

The higher occurrence of anti-*Brucella* spp. antibodies in small mammals on dairy farms could indicate that livestock species matter. However, cows and pigs carry different *Brucella* species, which was not considered because the bacteria could not be isolated. Natural *Brucella* spp. infections in rodents have not been explored in detail but deserve attention because they can transmit different *Brucella* species [i.e., 21, 48]. To our knowledge, this is the first study with evidence of natural *Brucella* spp. infection in small mammals in Argentina that explores the related intrinsic and extrinsic factors. The role of small mammals regarding *Brucella* spp. infection should be further explored. Higher seroprevalence in the winter and summer could be related to livestock births because *Brucella* spp. is shed during birth or abortion. Therefore, it would be necessary to include livestock infections in addition to small mammal infections in future work.

Only host abundance affected *Trichinella* spp. occurrence. Contrary to our hypothesis, we did not find differences between production systems at any level, which suggests that *Trichinella* spp. is dispersed on both dairy and pig farms and that the presence of pigs is not necessary for the infection of small mammals.

Metacestode occurrence was only related to host abundance at the community and *R*. *norvegicus* population levels. We are not aware of prior work considering metacestodes beyond the individual level. However, some authors found associations between temperature, humidity and density-dependent factors and helminth communities in rodents [65, 129, 130]. The correlation between *C*. *fasciolaris* infection in Norway rats and temperature indicates potential effects on the survival of *C*. *fasciolaris* eggs, as proposed by Deter *et al*. [130]. Individual infection was also influenced by age and habitat, as previously reported [65, 66, 130]. Because this parasite is indirectly transmitted, it is necessary to know the abundance and deposition behavior of the definitive hosts and egg survival per habitat to draw firm conclusions [130, 131].

Even if it is somewhat preliminary, this study substantially increases the previously slim knowledgebase of the ecology of small mammal-borne disease in livestock production systems. The findings can be used for more detailed future studies. Removal sampling in this study appeared to have no considerable effect on small mammal abundance [7], but the potential effects that it would have on pathogens were not considered. Future studies based on non-invasive methods (capture/recapture) should clarify this aspect.

Murines seem to be the most important species in the maintenance of pathogens on farms due to their high abundance [7]. They have small home ranges and are rarely found outside farms [132–134] and may therefore not be vital in spreading pathogens among farms. Opossums have larger home ranges [135] and may be a crucial link that carries pathogens to neighboring farms and other habitats. Several farms arranged as “islands” surrounded by crop fields and rangelands can be inside the home range of individual opossums. However, information about these relevant marsupials in agroecosystems in the Pampas region is scarce. *Akodon azarae* is also a carrier of some pathogens and *O*. *flavescens* carried *Leptospira* spp. Both sigmodontines are common in the agroecosystems that border farms. They may also maintain and distribute *Brucella* spp. and *Leptospira* spp. However, it is not known if the pathogen prevalence of populations in and around crops is similar to farm populations. The interaction among opossums spreading zoonoses and murines (especially Norway rats) and some sigmodontines maintaining zoonoses in animal production systems may create stable hotspots for particular diseases. Therefore, maintaining small mammals that are most likely to carry pathogens at low abundance, below the limit values identified in this study, may contribute to the prevention of zoonotic disease in these systems. We suggest focusing management efforts mainly on dwellings where murines are more abundant [7] but, undertaking rodent proofing to prevent re-entry, because the single use of chemical control creates dispersal sinks increasing the number of individuals that colonize the dwellings, thus potentially increasing the immigration of infected individuals [136]. Additionally, farm perimeter habitats should be managed to isolate them from their surroundings. Avoiding the presence of corridors such as weedy fences and channel vegetation may prevent the dispersal of opossums and other native small mammals among farms [137].

## Supporting information

S1 Appendix Summary of the Generalized Lineal Models (GLM) for each pathogen at the three studied levels. Summary of the Generalized Lineal Models (GLM) for each pathogen at the small mammal community level, and at the *Rattus norvegicus* population and individual level, on the 18 studied livestock farms of central Argentina from 2008 to 2011. SM AB: small mammal abundance (trap success: individuals/trap-nights); PP and PP6: accumulated monthly precipitation and accumulated precipitation in the last six months, respectively; Type: type of productive system (dairy or pig farm); Temp: monthly mean temperature (°C).
(DOC)
