# Supplementary material for: Intrinsic and extrinsic factors related to pathogen infection in wild small mammals in intensive milk cattle and swine production systems
Source: PLoS Negl Trop Dis. 2017 Jun 30;11(6):e0005722. doi: 10.1371/journal.pntd.0005722 (PMC5509364; doi:10.1371/journal.pntd.0005722)
Supplement: S1 Appendix — Summary of the Generalized Lineal Models (GLM) for each pathogen at the small mammal community level, and at the Rattus norvegicus population and individual level, on the 18 studied livestock farms of central Argentina from 2008 to 2011. SM AB: small mammal abundance (trap success: individuals/trap-nights); PP and PP6: accumulated monthly precipitation and accumulated precipitation in the last six months, respectively; Type: type of productive system (dairy or pig farm); Temp: monthly mean temperature (°C). (DOC) [file pntd.0005722.s001.doc]

**S1 Appendix. Summary of the Generalized Lineal Models (GLM) for each pathogen at the three studied levels.**

Summary of the Generalized Lineal Models (GLM) for each pathogen at the small mammal community level, and at the *R. norvegicus* population and individual level, on the 18 studied livestock farms of central Argentina from 2008 to 2011. SM AB: small mammal abundance (trap success: individuals/trap-nights); PP and PP6: accumulated monthly precipitation and accumulated precipitation in the last six months, respectively; Type: type of productive system (dairy or pig farm); Temp: monthly mean temperature (°C).

| ***Leptospira* spp.** | |  |  |
| --- | --- | --- | --- |
|  |  |  |  |
| **Community level** |  |  |  |
| **Ocurrence:** ~ SM AB |  |  |  |
|  |  |  |  |
| Explanatory variable | Estimate ± SE | *z* value | *p* |
| Intercept | -0.788 ± 0.431 | -1.830 | 0.067 |
| SM AB | 10.584 ± 4.471 | 2.367 | 0,018 |
|  |  |  |  |
| **Population level** |  |  |  |
| ***R. norvegicus* ocurrence:** ~ PP * SM AB |  |  |  |
|  |  |  |  |
| Explanatory variable | Estimate ± SE | *z* value | *p* |
| Intercept | 0.053 ± 0.816 | 0.065 | 0.9484 |
| PP | -1.530 ± 1.025 | -1.492 | 0.136 |
| SM AB | -6.029 ± 7.874 | -0.766 | 0.444 |
| PP * SM AB | 29.113 ± 17.167 | 1.696 | 0.090 |
|  |  |  |  |
| ***R. norvegicus* prevalence:** ~ PP * SM AB |  |  |  |
|  |  |  |  |
| Explanatory variable | Estimate ± SE | *z* value | *p* |
| Intercept | -0.085 ± 0.455 | -0.186 | 0.852 |
| PP | -0.831 ± 0.394 | -2.109 | 0.035 |
| SM AB | -11.800 ± 3.544 | -3.330 | <0.001 |
| PP * SM AB | 14.600 ± 4.580 | 3.188 | 0.001 |
| **Individual level** |  |  |  |
| ***R. norvegicus* infection:** ~ Season* PP6 |  |  |  |
|  |  |  |  |
| Explanatory variable | Estimate ± SE | *z* value | *p* |
| Intercept | 2.005 ± 1.591 | 1.260 | 0.208 |
| Season Spring | -4.537 ± 2.548 | -1.781 | 0.075 |
| Season Summer | -2.867 ± 1.672 | -1.714 | 0.086 |
| Season Winter | -1.556 ± 2.227 | -0.698 | 0.485 |
| PP6 | -0.813 ± 0.347 | -2.343 | 0.019 |
| Season Spring * PP6 | 1.092 ± 0.557 | 1.961 | 0.050 |
| Season Summer * PP6 | 0.860 ± 0.358 | 2.403 | 0.016 |
| Season Winter * PP6 | 0.363 ± 0.551 | 0.659 | 0.510 |
|  |  |  |  |
| ~ Habitat ^(1)^ * PP6 |  |  |  |
|  |  |  |  |
| Explanatory variable | Estimate ± SE | *z* value | *p* |
| Intercept | 0.565 ± 0.766 | 0.738 | 0.460 |
| Habitat Drainage channels | -2.568 ± 1.107 | -2.320 | 0.020 |
| Habitat Food sheds | 0.233 ± 1.345 | 0.174 | 0.862 |
| Habitat Human buildings | -6.237 ± 2.431 | -2.566 | 0.010 |
| PP6 | -0.301 ± 0.177 | -1.697 | 0.090 |
| Habitat Drainage channels * PP6 | 0.398 ± 0.228 | 1.746 | 0.081 |
| Habitat Food sheds * PP6 | -0.274 ± 0.351 | -0.782 | 0.434 |
| Habitat Human buildings * PP6 | 0.970 ± 0.347 | 2.799 | 0.005 |

*^(1)^ Animal sheds, drainage channels, food sheds and human buildings. Vegetated areas could not be analyzed.*

| **Anti - *Brucella* spp. antibodies** | | |  | | |  | |
| --- | --- | --- | --- | --- | --- | --- | --- |
|  | |  |  | | |  | |
| **Community level** | |  |  | | |  | |
| **Ocurrence:** ~ SM AB + Season | |  |  | | |  | |
|  | |  |  | | |  | |
| Explanatory variable | Estimate ± SE | | | *z* value | | *p* | |
| Intercept | -3.247 ± 1.052 | | | -3.086 | | 0.002 | |
| SM AB | 21.553 ± 6.608 | | | 3.262 | | 0.001 | |
| Season Spring | -0.228 ± 1.196 | | | -0.191 | | 0.848 | |
| Season Summer | 1.324 ± 1.037 | | | 1.276 | | 0.202 | |
| Season Winter | 2.286 ± 1.048 | | | 2.182 | | 0.029 | |
|  |  | | |  | |  | |
| ~ SM AB * Type |  | | |  | |  | |
|  |  | | |  | |  | |
| Explanatory variable | Estimate ± SE | | | *z* value | | *p* | |
| Intercept | -1.370 ± 0.638 | | | -2.148 | | 0.032 | |
| SM AB | 7.491 ± 6.857 | | | 1.092 | | 0.275 | |
| Type Dairy farms | -1.594 ± 1.297 | | | -1.229 | | 0.219 | |
| SM AB * Type Dairy farms | 24.478 ± 13.844 | | | 1.768 | | 0.077 | |
|  |  | | |  | |  | |
| **Population level** |  | | |  | |  | |
| ***R. norvegicus* ocurrence:** ~ SM AB |  | | |  | |  | |
|  |  | | |  | |  | |
| Explanatory variable | Estimate ± SE | | | *z* value | | *p* | |
| Intercept | -2.675 ± 0.801 | | | -3.340 | | 0.001 | |
| SM AB | 24.107 ± 7.727 | | | 3.120 | | 0.002 | |
|  | |  |  | | |  | |
| ***R. norvegicus* prevalence:** ~ Season | |  |  | |  | |  |
|  | |  |  | |  | |  |
| Explanatory variable | Estimate ± SE | | | *z* value | | *p* | |
| Intercept | -1.992 ± 0.435 | | | -4.578 | | <0.001 | |
| Season Spring | 0.571 ± 0.527 | | | 1.083 | | 0.279 | |
| Season Summer | 1.045 ± 0.512 | | | 2.040 | | 0.041 | |
| Season Winter | 1.636 ± 0.520 | | | 3.146 | | 0.002 | |
|  | |  |  | |  | |  |
| **Individual level** | |  |  | |  | |  |
| ***R. norvegicus* infection:** ~ Season + Habitat ^(2)^ * Type + Habitat ^(2)^ * SM AB | | | | | | |  |
|  | |  |  | |  | |  |
| Explanatory variable | Estimate ± SE | | | *z* value | | *p* | |
| Intercept | -1.259 ± 0.829 | | | -1.520 | | 0.129 | |
| Season Spring | 0.377 ± 0.601 | | | 0.628 | | 0.530 | |
| Season Summer | 1.450 ± 0.605 | | | 2.397 | | 0.017 | |
| Season Winter | 1.946 ± 0.619 | | | 3.145 | | 0.002 | |
| Habitat Drainage channels | -2.405 ± 1.130 | | | -2.129 | | 0.033 | |
| Habitat Food sheds | -3.215 ± 2.404 | | | -1.337 | | 0.181 | |
| Habitat Vegetated areas | -8.316 ± 5.939 | | | -1.400 | | 0.161 | |
| Type Pig farms | -1.901 ± 0.911 | | | -2.088 | | 0.037 | |
| SM AB | -3.080 ± 3.264 | | | -0.944 | | 0.345 | |
| Habitat Drainage channels * Type Pig farms | 2.884 ± 1.115 | | | 2.586 | | 0.010 | |
| Habitat Food sheds * Type Pig farms | 2.143 ± 2.090 | | | 1.026 | | 0.305 | |
| Habitat Vegetated areas * Type Pig farms | 3.571 ± 2.511 | | | 1.422 | | 0.155 | |
| Habitat Drainage channels * SM AB | 10.292 ± 5.091 | | | 2.022 | | 0.043 | |
| Habitat Food sheds * SM AB | 13.539 ± 8.117 | | | 1.668 | | 0.095 | |
| Habitat Vegetated areas * SM AB | 47.789 ± 31.168 | | | 1.533 | | 0.125 | |

*^(2)^ Animal sheds, drainage channels, food sheds and vegetated areas. Human buildings could not be analyzed.*

| ***Trichinella* spp.** | | | | |  | | |  | |  |
| --- | --- | --- | --- | --- | --- | --- | --- | --- | --- | --- |
|  | |  | | |  | | |  | |  |
| **Community level** | |  | | |  | | |  | |  |
| **Ocurrence:** ~ SM AB | |  | | |  | | |  | |  |
|  | |  | | |  | | |  | | |
| Explanatory variable | Estimate ± SE | | | *z* value | | | *p* | | |  |
| Intercept | -2.165 ± 0.518 | | | -4.181 | | | <0.001 | | |  |
| SM AB | 11.622 ± 3.995 | | | 2.909 | | | 0.004 | | |  |
|  | |  | | |  | | |  | |  |
| **Population level** | |  | | |  | | |  | |  |
| ***R. norvegicus* occurrence:** ~ SM AB | |  | | |  | | |  | |  |
|  | |  | | |  | | |  | |  |
| Explanatory variable | Estimate ± SE | | | *z* value | | | *p* | | |  |
| Intercept | -3.347 ± 0.870 | | | -3.846 | | | <0.001 | | |  |
| SM AB | 12.812 ± 4.939 | | | 2.594 | | | 0.009 | | |  |
|  | |  | | |  | | |  | |  |
| **Individual level** | |  | | |  | | |  | |  |
| ***R. norvegicus* infection:** ~ Age * Sex | |  | | |  | | |  | |  |
|  | |  | | |  | | |  | |  |
| Explanatory variable | Estimate ± SE | | | *z* value | | | *p* | | |  |
| Intercept | -5.543 ± 1.395 | | | -3.972 | | | <0.001 | | |  |
| Age | 0.235 ± 0.140 | | | 1.681 | | | 0.092 | | |  |
| Sex Male | 3.403 ± 1.568 | | | 2.171 | | | 0.030 | | |  |
| Age * Sex Male | -0.510 ± 0.244 | | | -2.093 | | | 0.036 | | |  |
|  | |  | | |  | | |  | |  |
| **Metacestodes** | |  | | |  | | |  | |  |
|  | |  |  | | |  | | |  | |
| **Community level** | |  |  | | |  | | |  | |
| **Ocurrence:** ~ SM AB | |  |  | | |  | | |  | |
|  | |  |  | | |  | | |  | |
| Explanatory variable | Estimate ± SE | | | *z* value | | | *p* | | |  |
| Intercept | -1.907 ± 0.534 | | | -3.569 | | | <0.001 | | |  |
| SM AB | 24.257 ± 7.304 | | | 3.321 | | | 0.001 | | |  |
|  | |  |  | | |  | | |  | |
| **Population level** | |  |  | | |  | | |  | |
| ***R. norvegicus* ocurrence:** ~ SM AB | |  |  | | |  | | |  | |
|  | |  |  | | |  | | |  | |
| Explanatory variable | Estimate ± SE | | | *z* value | | | *p* | | |  |
| Intercept | -1.776 ± 0.745 | | | -2.383 | | | 0.017 | | |  |
| SM AB | 32.187 ± 11.812 | | | 2.725 | | | 0.006 | | |  |
|  | |  |  | | |  | | |  | |
| ***R. norvegicus* prevalence:** ~ Type + PP6 | |  |  | | |  | | |  | |
|  | |  |  | | |  | | |  | |
| Explanatory variable | Estimate ± SE | | | *z* value | | | *p* | | |  |
| Intercept | -0.363 ± 0.337 | | | -1.078 | | | 0.281 | | |  |
| Type Dairy farms | 1.018 ± 0.351 | | | 2.900 | | | 0.004 | | |  |
| PP6 | -0.168 ± 0.089 | | | -1.878 | | | 0.060 | | |  |
|  | |  |  | | |  | | | |  |
| ~ SM AB | |  |  | | |  | | | |  |
|  | |  |  | | |  | | | |  |
| Explanatory variable | Estimate ± SE | | | *z* value | | | *p* | | |  |
| Intercept | -1.008 ± 0.241 | | | -4.188 | | | <0.001 | | |  |
| SM AB | 3.169 ± 1.148 | | | 2.760 | | | 0.006 | | |  |

|  | **Individual level** | | | | | | |
| --- | --- | --- | --- | --- | --- | --- | --- |
|  | ***R. norvegicus* infection:** ~ SM AB * Age + Habitat * Type + Temp. | | | | | | |
|  | | |  | |  | |  |
| Explanatory variable | | Estimate ± SE | | *z* value | | *p* | |
| Intercept | | 0.540 ± 0.765 | | 0.706 | | 0.480 | |
| Age | | -0.074 ± 0.083 | | -0.888 | | 0.375 | |
| SM AB | | -6.198 ± 2.978 | | -2.081 | | 0.037 | |
| Habitat Drainage channels | | -0.383 ± 0.457 | | -0.836 | | 0.403 | |
| Habitat Food sheds | | 0.762 ± 0.589 | | 1.293 | | 0.196 | |
| Habitat Human buildings | | -1.906 ± 0.854 | | -2.232 | | 0.026 | |
| Habitat Vegetated areas | | -1.548 ± 0.983 | | -1.575 | | 0.115 | |
| Type Pig farms | | -1.531 ± 0.728 | | -2.102 | | 0.036 | |
| Temp. | | -0.065 ± 0.030 | | -2.193 | | 0.028 | |
| Age * SM AB | | 2.205 ± 0.512 | | 4.305 | | 0.000 | |
| Hábitat Drainage channels * Type Pig farms | | 2.871 ± 0.893 | | 3.214 | | 0.001 | |
| Hábitat Food sheds * Type Pig farms | | 0.192 ± 0.989 | | 0.195 | | 0.846 | |
| Hábitat Human buildings * Type Pig farms | | 4.148 ± 1.489 | | 2.785 | | 0.005 | |
| Hábitat Vegetated areas * Type Pig farms | | 2.356 ± 1.365 | | 1.726 | | 0.084 | |
